# Sylvatic Dengue Virus Type 2 Activity in Humans, Nigeria, 1966

**DOI:** 10.3201/eid1403.070843

**Published:** 2008-03

**Authors:** Nikos Vasilakis, Robert B. Tesh, Scott C. Weaver

**Affiliations:** * University of Texas Medical Branch, Galveston, Texas, USA; 1 Current affiliation: University of Pittsburgh, Pittsburgh, Pennsylvania, USA.

**Keywords:** Sylvatic dengue, arbovirus, flavivirus, West Africa, phylogenetic analysis, dispatch

## Abstract

Using phylogenetic analysis of complete virus genomes from human isolates obtained in Nigeria in 1966, we identified sylvatic dengue virus (DENV) strains from 3 febrile patients. This finding extends current understanding of the role of sylvatic DENV in febrile disease and documents another focus of sylvatic DENV transmission in West Africa.

Sylvatic dengue viruses (DENVs) are ecologically and evolutionary distinct ancestral lineages that circulate in forests of Southeast Asia and West Africa between nonhuman primates and arboreal *Aedes* mosquitoes. In Asia, serologic analysis and virus isolations suggest an association between *Macaca* spp. and *Presbytis* spp. monkeys and sylvatic DENV-1, -2, and -4, with *Aedes niveus* as the principal vector (*1*). Sylvatic DENV-3 has not been isolated, but it is believed to exist in Malaysia on the basis of sentinel monkey seroconversions (*1*). In West Africa, only sylvatic DENV-2 has been identified, where it circulates among *Erythrocebus patas* monkeys and sylvatic mosquitoes, including *Ae*. *furcifer*, *Ae*. *vitattus*, *Ae*. *taylori*, and *Ae*. *luteocephalus* (*2*–*6*).

Sylvatic DENV strains are considered the evolutionary progenitors of endemic and epidemic (henceforth called endemic) strains that are transmitted among humans in urban environments throughout the tropics and subtropics by the peridomestic mosquitoes *Ae*. *aegypti* and *Ae*. *albopictus*. Previously, no evidence has shown that sylvatic DENV cycles are involved in outbreaks of human dengue, which involve the genetically and ecologically distinct endemic strains. Available data suggest that sylvatic strains are either confined to forest habitats or produce relatively mild illness, as described in a few documented human sylvatic DENV-2 infections in West Africa (*5*,*6*). Additionally, the small number of documented human infections suggests that sylvatic DENV-2 strains do not produce secondary human infections (spillover epidemics). One possible explanation for confinement of sylvatic DENV strains to the forest is that they do not have contact with the peridomestic vectors *Ae*. *aegypti* and *Ae*. *albopictus*, which are not abundant in enzootic regions.

Recent reports have shown that the gallery forest–dwelling mosquito *Ae*. *furcifer* is highly susceptible to sylvatic DENV infection (*2*) and disperses from the forest into villages in eastern Senegal (*3*). This finding suggests that this species may act as a bridge vector for exchange between forest and peridomestic habitats. Furthermore, the ability of *Ae*. *aegypti* and *Ae*. *albopictus* to transmit sylvatic DENV (*2*), as well as the lack of evidence that any adaptation of sylvatic DENV is needed to replicate efficiently in humans (*7*), suggests that transfer between forest and human habitats could occur regularly.

## The Study

Given the above evidence, we hypothesized that unrecognized spillover epidemics may be caused by sylvatic DENV-2 strains in West Africa. We therefore examined isolates of DENV recovered from febrile patients, ranging from 3 months to 38 years of age, who were seen at the outpatient department of the University College Hospital, Ibadan, Nigeria, from August 1964 through December 1968 (*8*). Complement fixation and neutralization tests (with the Hawaii strain of DENV-1 and the Trinidad-1751 strain of DENV-2 as reference strains) classified 14 of 32 original isolates as DENV-2 (*8*). Three of these 14 isolates were obtained from the University of Texas Medical Branch World Reference Center for Emerging Viruses and Arboviruses; the other 11 are not known to exist in any virus collection (Table).

**Table:** History of dengue virus type 2 isolates collected from patients with febrile illness in Ibadan, Nigeria, 1964–1968*

Isolate†	Patient age/sex	Source	Passage history‡	Date of collection	GenBank accession no.
IBH319	3 mo/F	Blood	NA	1964 Aug 11	–
IBH10126	8 y/M	Blood	NA	1966 Jun 1	–
IBH11208§	3.5 y/F	Blood	SM5, C6/36 –1	1966 Aug 17	EF105307
IBH11234§	31 y/M	Blood	SM16, C6/36 –1	1966 Aug 18	EU003591
IBH11358	5 mo/F	Blood	NA	1966 Aug 24	–
IBH11444	6 y/F	Blood	NA	1966 Aug 31	–
IBH11449	1.5 y/F	Blood	NA	1966 Aug 31	–
IBH11664§	1.5 y/M	Blood	SM30, C6/36 –1	1966 Sep 12	EF105388
IBH11935A	13 y/M	Blood	NA	1966 Sep 23	–
IBH12541	23 y/M	Serum	NA	1966 Oct 17	–
IBH13028A	24 y/M	Serum	NA	1966 Nov 17	–
IBH24075	1.5 y/F	Blood	NA	1968 Jan 12	–
IBH26489A	5 y/M	Blood	NA	1968 Apr 24	–
IBH26953	3 y/M	Blood	NA	1968 May 15	–

*Available dengue virus isolates were obtained from the University of Texas Medical Branch World Reference Center for Emerging Viruses and Arboviruses. †A complete inventory of dengue viruses isolated in Ibadan during 1964–1969 is described in the 1969 annual report of the University of Ibadan, Arbovirus Research Project, p. 152–7. ‡NA, not available; SM, suckling mouse; C6/36, *Aedes albopictus* cell line. –1 indicates that the virus isolates were passaged once in C6/36 cultures in our laboratory to obtain high-titer stocks. §Virus isolates used in this study.

RNA was extracted from these 3 isolates, after passage in C6/36 mosquito cells, by using the QIAamp Viral RNA Mini Kit (QIAGEN, Valencia, CA, USA). Their complete genomic sequences were determined by designing overlapping PCR amplicons. After purification by electrophoresis on 1% agarose gels, both strands were sequenced directly by using the ABI (Roche Diagnostics, Indianapolis, IN, USA) protocols with both the PCR and internal primers to derive a consensus sequence. We then generated a phylogenetic tree, including 22 endemic DENV-2 isolates representing strains from diverse localities throughout the tropics and neotropics, as well as representative strains of DENV-1, DENV-4 (including sylvatic isolates P72–1244 and P75–215, respectively), and DENV-3 strains as outgroups (41 sequences, 10,185 nt in length). Phylogenetic trees were inferred by using Bayesian analysis with 1 million iterations (*9*) as well as maximum parsimony and neighbor-joining implemented in PAUP version 4.0 (*10*) (Figure).

**Figure:**
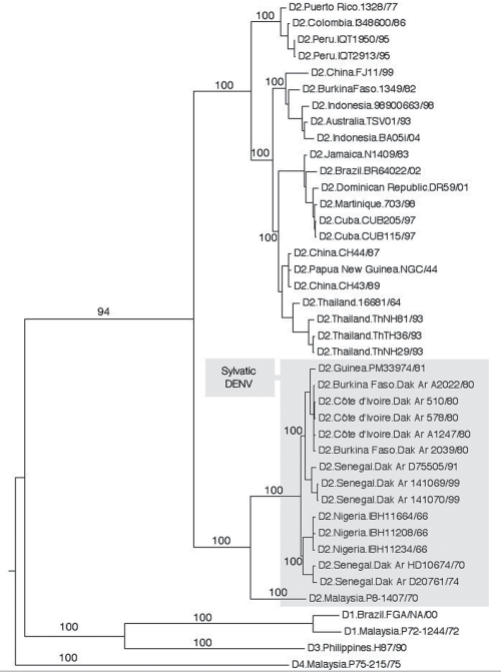
Phylogenetic relationships of 3 complete coding regions of Nigerian dengue virus type 2 (DENV-2) isolates obtained from febrile patients in Ibadan, Nigeria, during the 1966 rainy season. A total of 15 sylvatic DENV-2 genomes (shaded) were compared with human isolates of DENV-2 and representatives of DENV-1, DENV-3, and DENV-4. Phylogeny was inferred by using Bayesian analysis, and all horizontal branches are scaled according to the number of substitutions per site. Bootstrap values are shown for key nodes.

All analyses indicated that all 3 Nigerian isolates were genetically distinct from endemic DENV-2 isolates, including those from West Africa, and fell within the sylvatic DENV-2 clade (Figure). Among sylvatic isolates, Malaysian and West African sylvatic DENV-2 strains were genetically distinct. All analyses also delineated a chronologic divide among isolates within the sylvatic African DENV-2 clade; all pre-1980 isolates formed a group distinct from all post-1980 isolates. This observation supports recent evidence of rapid sylvatic DENV turnover caused by high nucleotide substitution rates (*11*). These findings also extend the temporal and spatial range in which sylvatic DENV are known to circulate in West Africa. The first documented isolation of sylvatic DENV in West Africa was from a febrile patient in Senegal identified in 1970 (*6*); the sylvatic isolates from the Nigerian patients predate this Senegalese strain by 4 years.

## Conclusions

The 1964–1968 DENV-2 activity among humans in Ibadan is epidemiologically interesting. Although we were able to obtain only 3 of the of 10 DENV-2 strains collected in 1966 (Table), their identification as sylvatic strains and isolation from febrile patients suggest the first documented outbreak of sylvatic DENV-2 in humans. We define outbreak as a small, localized group of affected persons; such groups are often confined to a village or a small geographic area. Although no written records on the location of residence or exposure of these patients exist, all resided within the Ibadan city limits (D.E. Carey, pers. comm.). At that time, humans and monkeys living within 4 diverse ecologic zones (rainforest, derived savannah, southern Guinea savannah, and plateau) in Nigeria showed high levels of DENV-2 neutralizing antibodies, which suggested enzootic/endemic DENV-2 and a sylvatic cycle in Nigeria 30 years ago (*12*,*13*). Furthermore, the clinical manifestation of dengue caused by these Nigerian sylvatic DENV-2 strains was indistinguishable from classic dengue fever caused by endemic strains (D.E. Carey, pers. comm.) (*5*,*6*,*14*). Isolation of sylvatic DENV-2 from febrile patients in an urban environment (*8*), the ability of peridomestic *Aedes* spp. mosquitoes (*Ae*. *aegypti* and *Ae*. *albopictus)* to serve as vectors for similar West African strains (*2*), and the lack of evidence that any adaptation of sylvatic DENV is needed to replicate efficiently in humans (*7*) suggest that spillover epidemics occur in urban settings.

Currently, limited availability of reliable epidemiologic information and inability to differentiate clinically or serologically between urban and sylvatic DENV-2 infection prevent an accurate assessment of the true extent of human exposure in West Africa. Thus, further study is needed to elucidate the interactions of sylvatic and urban transmission. Even with functional reporting and surveillance, clinical diagnosis of dengue in West Africa is complicated by cocirculation of several other viruses that cause clinically similar febrile diseases (e.g., chikungunya, o’nyong-nyong, Zika viruses) (*15*). Other obstacles to accurate assessment of the public health effect of sylvatic DENV in Africa include limited access of the population to healthcare facilities, lack of access to viral and serologic diagnostics, popular beliefs that discourage standard medical treatment unless illness is severe, and a lack of surveillance of monkeys. To overcome these obstacles, comprehensive ecologic and epidemiologic studies are needed to assess the roles of nonhuman primates and other vertebrate hosts in the maintenance of sylvatic DENV, the degree and routes of ecologic contact between humans and sylvatic DENV, and the replication and immunologic dynamics of sylvatic DENV in nonhuman primates.
